# Implications for workability and survivability in populations exposed to extreme heat under climate change: a modelling study

**DOI:** 10.1016/S2542-5196(18)30240-7

**Published:** 2018-12

**Authors:** Oliver Andrews, Corinne Le Quéré, Tord Kjellstrom, Bruno Lemke, Andy Haines

**Affiliations:** a Tyndall Centre for Climate Change Research, School of Environmental Sciences, University of East Anglia, Norwich, UK; b School of Geographical Sciences, University of Bristol, Bristol, UK; c Center for Technology Research and Innovation, Limassol, Cyprus; d School of Demography, Australian National University, Canberra, ACT, Australia; e School of Health, Nelson Marlborough Institute of Technology, Nelson, New Zealand; f Department of Public Health, Environments, and Society and Department of Population Health, London School of Hygiene & Tropical Medicine, London, UK

## Abstract

**Background:**

Changes in temperature and humidity due to climate change affect living and working conditions. An understanding of the effects of different global temperature changes on population health is needed to inform the continued implementation of the Paris Climate Agreement and to increase global ambitions for greater cuts in emissions. By use of historical and projected climate conditions, we aimed to investigate the effects of climate change on workability (ie, the ability to work) and survivability (the ability to survive).

**Methods:**

In this modelling study, we estimated the changes in populations exposed to excessive heat stress between the recent past (ie, 1986–2005) and 2100. We used climate data from four models to calculate the wet-bulb globe temperature, an established heat exposure index that can be used to assess the effects of temperature, humidity, and other environmental factors on humans. We defined and applied thresholds for risks to workability (where the monthly mean of daily maximum wet-bulb globe temperature exceeds 34°C) and survivability (where the maximum daily wet-bulb globe temperature exceeds 40°C for 3 consecutive days), and we used population projections to quantify changes in risk associated with different changes to the global temperature.

**Findings:**

The risks to workability increase substantially with global mean surface temperature in all four climate models, with approximately 1 billion people affected globally after an increase in the global temperature of about 2·5°C above pre-industrial levels. There is greater variability between climate models for exposures above the threshold for risks to survivability than for risks to workability. The number of people who are likely to be exposed to heat stress exceeding the survivability threshold increases with global temperature change, to reach around 20 million people globally after an increase of about 2·5°C, estimated from the median of the models, but with a large model uncertainty. More people are likely to be exposed to heat stress in urban than in rural areas. Population exposure can fluctuate over time and change substantially within one decade.

**Interpretation:**

Exposure to excessive heat stress is projected to be widespread in tropical or subtropical low-income and middle-income countries, highlighting the need to build on the Paris Agreement regarding global temperature targets, to protect populations who have contributed little to greenhouse gas emissions. The non-linear dependency of heat exposure risk on temperature highlights the importance of understanding thresholds in coupled human-climate systems.

**Funding:**

Wellcome Trust.

## Introduction

The Conference of the Parties (COP), a body of the UN, met in Paris in 2015 (COP 21), and produced an international agreement on climate change that provides a step towards the effective protection of planetary health. The Paris Agreement states that the global community needs to reduce greenhouse gas emissions so that the mean global temperature change (GTC) can remain well below a 2°C increase above pre-industrial levels, and that it needs to pursue efforts to limit the temperature increase to 1·5°C above pre-industrial levels. However, the combined effect of the nationally determined contributions, representing the pledged mitigation of greenhouse gas emissions that countries submitted in advance of COP21, would result in an estimated GTC of around 3°C by 2100,^1^ and a higher GTC if mitigation measures fail.

Exposure to high temperatures can cause heat exhaustion and heat stroke,^2^ leading to aggregate effects that could reduce labour productivity^3^, ^4^ and increase the risk of temperature-associated mortality.^5^ An increase in global summertime heat stress on humans between 1973 and 2012 has been reported.^6^ Future increases in heat stress have been projected over the next few decades at the regional^7^ and global^8^ scales in response to increasing concentrations of greenhouse gases. However, it is important to understand how projected warming that meets the international commitment to limit the GTC well below 2°C might affect population health^9^ and what the consequences for failing to meet this target might be. By use of historical and projected climate conditions, we aimed to investigate the effects of climate change on workability (ie, the ability to work) and survivability (the ability to survive), which may have implications for the potential habitability of heat-vulnerable regions.

**Research in context**

**Evidence before this study**

An important feature of global climate change is increasing air temperatures across most areas of the world. Extremes of atmospheric temperature are a known health hazard, since good health relies on maintaining the core body temperature within a narrow range (36·5–37·5°C) under different external environmental conditions. If core body temperature increases to more than 38–39°C, there is a risk of heat exhaustion. At core body temperatures of more than 40°C, serious heat stroke can cause death. Evaporation of sweat is a key mechanism for cooling the body when the external temperature is above 35°C. In high-humidity environments, evaporation of sweat is inhibited, so this important natural heat loss mechanism is undermined. Many tropical and subtropical areas can already experience high levels of heat stress annually for several months of the year. Ongoing climate change is projected to substantially increase temperatures in many densely populated areas. In assessments of the health risks associated with increasing global temperature, it is essential to consider temperature, humidity, and the heat generated from physical activity. Acclimatisation to high temperature does occur, but there is a limit, and combinations of high temperature and high humidity can lead core temperature to reach problematic levels.

**Added value of this study**

Most epidemiological studies and impact assessments have analysed the effects of high temperatures on mortality during heat waves and have focused on older people. In this study, we analysed the effects of temperature, humidity, and work rate on adults in their daily activities (workability) and on their physiological ability to cope with heat (survivability). By use of bias-corrected climate model data to calculate the wet-bulb globe temperature, the most widely used heat-stress index used to assess health risks in physical work situations, we found that risks to workability and survivability increase with modelled global temperature changes, particularly in tropical or subtropical regions. To our knowledge, no previous peer-reviewed publication has presented such an analysis, which projects changes in heat exposure risk based on thresholds for work and survival, and which contributes to the accumulating evidence of the serious planetary health threats of climate change.

**Implications of all the available evidence**

In combination with previous reports, our assessment of health effects highlights the importance of environmental heat for global population health. The heat stress that we have evaluated affects the active adult population in many countries during the hot season, and it is not just restricted to those experiencing extreme heat waves. During the hot season, people are already physically acclimatised and adaptation via air conditioning in residences and workplaces can reduce heat exposure risk. However, it is unlikely that people receiving low incomes will be able to afford efficient cooling systems and many work situations cannot always be protected with such systems. In many cases, those affected by extreme heat will receive greatly reduced income for at least 1 month a year, which will increase the risk of impoverishment. This new evidence on the effects of climate change should inform future policies for the protection of planetary health via mitigation of climate change, highlighting the importance of limiting global temperature increases in line with Paris Agreement targets.

## Methods

### Study design

In this modelling study, we analysed the risk of heat exposure in humans at global scales, combining climate change projections for this century (ie, from the recent past [1986–2005] to 2100) from several current climate models with existing population projections. We used these combined climate–population scenarios to project changes to workability and survivability that could occur in response to GTC targets, based on recently proposed heat stress thresholds.

### Climate model data

Heat exposure risk is quantified with the wet-bulb globe temperature (WBGT), which is based on the measurable meteorological quantities of air temperature, humidity, total heat radiation, and air movement (wind speed).

In this analysis, we used phase 5 of the Coupled Model Intercomparison Project (CMIP5)^10^ to project changes in heat stress by 2100 relative to those of the recent past (1986–2005). Simulations were provided by phase 2b of the Inter-Sectoral Impact Model Intercomparison Project (ISIMIP2b).^11^ We focused on the climate projection by following the Representative Concentration Pathway 6.0 (RCP6.0), which represents a mid-range climate future^12^ from which it is possible to investigate a range of GTCs. We used all four CMIP5 models included in ISIMIP2b (GFDL-ESM2M, HadGEM2-ES, IPSL-CM5A-LR, and MIROC5). This ensemble has been shown to cover an equivalent fractional uncertainty range when compared with other randomly chosen four-member sets of CMIP5 models.^11^

Model variables were independently bias-adjusted towards observations from a 2016 reference dataset (EWEMBI).^13^ To adjust for bias, model variables were remapped onto a 0·5° regular grid with a first-order conservative scheme that preserved spatial averages. Bias adjustment to closely align model results with observations is necessary when applying temperature and humidity in studies of human effects that require absolute values rather than anomalies. The ISIMIP2b bias adjustment approach was previously described^11^ and includes important modifications to the ISIMIP fast-track method,^14^ such as newly developed corrections for humidity. Bias adjustment was applied to both the model mean state and its variability.

We calculated the global WBGT by following the approach of Bernard and Pourmoghani,^15^ which has been shown to be an accurate estimator of WBGT in shaded conditions or conditions indoors without cooling.^16^ In accordance with the study by Hyatt and colleagues,^17^ we assumed a constant wind speed of 1 m/s when simulating air movement over skin during moderate physical activity (appendix) and no radiation sources. In-shade WBGT was used to represent a metric of unavoidable heat stress, as distinct from models of outdoor in-sun WBGT, which include the additional effects of total heat radiation.^18^ WBGT was calculated from air temperature, vapour pressure, and barometric surface pressure (appendix) with daily climate data, to simulate patterns of acute heat exposure. WBGT was estimated for the warmest part of the day by use of daily maximum surface air temperature. Vapour pressure was calculated from the ISIMIP2b daily mean specific humidity and barometric surface pressure, and dewpoint temperature was calculated from vapour pressure (appendix). Atmospheric moisture content changes derived from specific humidity show only a small diurnal component when compared with other metrics such as relative humidity, which depends on temperature and therefore has large diurnal variation. Additionally, we calculated estimates of GTC for each model from simulated land-ocean global mean surface temperature as decadal averages.

We used decadal population data^19^ (for 2010–2100) to project changes in population density under Shared Socioeconomic Pathway 2 (SSP2). By use of the scenario matrix approach of Van Vuuren and colleagues,^20^ we used the central SSP2 population projection for this century because of its overall compatibility with the RCP6.0 climate scen·ario in terms of adaptation and mitigation policies. Population data were stratified to include rural and urban populations with a gravity model-based approach.^19^ The model was calibrated with historical data, and the SSP narrative was interpreted to specify internally consistent spatial patterns of urban and rural development.

### Heat stress thresholds

Mechanisms for thermoregulation of the human body and the physical responses to dangerous heat exposure are well understood. Although adaptation to present and future climate-driven heat stress is likely to result in varying population responses based on income level and other social factors, physiological thermodynamic arguments suggest that upper limits to adaptation could affect the survival of individuals.^21^ In the absence of reliable active cooling measures, such as air conditioning, extreme heat threatens the habitability of some regions as a result of inability to perform essential activities of daily living, such as physical work, when individual exposure thresholds are reached. Exposure to climate-change driven heat stress might have already caused rural to urban human migration in Pakistan.^22^

To describe the spatial distribution of heat stress around the world, we used three thresholds linked to international^23^ and national^24^ heat stress protection recommendations for working people. These risk thresholds are reached when the monthly mean of daily maximum WBGT in the hottest month exceeds 26°C (moderate), 30°C (high), or 34°C (extreme) in non-cooled workplaces.^17^

The effects of heat exposure accumulate depending on the duration and persistence of the exposure. We used two thresholds that estimate aggregated daily heat exposure risks, which were adapted from the proposals of Kjellstrom and colleagues^25^ and broadly reflect the ability to work (workability) and the ability to survive for heat-sensitive people (survivability). These thresholds reflect current work safety measures with an additional 3-day persistence criterion for the survivability threshold, based on heatwave duration studies.^26^, ^27^ These thresholds refer to shaded conditions without adaptation (eg, active cooling) and are therefore particularly relevant for countries where part of the economy relies on work in workshops and factories without efficient cooling.

We defined the upper threshold for workability as a monthly mean of the daily WBGT during the warmest part of the day of 34°C. For work that causes a metabolic rate of 300 W, in which the monthly mean WBGT for the hottest part of the day exceeds 34°C, it is suggested that it is too hot to work safely for a large part of the month.^25^ This workability threshold is supported by the recommended heat exposure limits of the US National Institute of Occupational Safety and Health^25^ for acclimatised individuals doing moderate activity work and corresponds to the limit at which the hourly capacity to work is reduced by at least 50% in field studies.^28^ Exceedance of this threshold implies severe disruption to working practices and has serious implications for livelihoods and increased risk of impoverishment.

The survivability threshold is defined as a heat stress condition in which exposure causes core body temperature to increase to potentially fatal levels during low-intensity physical activity.^25^ This threshold was set as daily maximum WBGT that exceeded 40°C for 3 consecutive days. This threshold builds on a previous study,^25^ but introduces the use of 3 consecutive days to provide a conservative estimate and limit the effects of avoidance strategies and adaptation, which could occur over longer periods of intermittent exposure. Increases in mortality rates during current heatwaves are related to both duration and intensity of exposure^26^, ^27^ but largely affect older people. Our proposed survivability limit is much higher than current exposures and would threaten exposed people of all ages. In our study, we used Climate Data Operators version 1.7, pyFerret version 1.2.0, and Ferret version 6.93 software.

### Role of the funding source

The funder of the study had no role in study design, data collection, data analysis, data interpretation, or writing of the report. The corresponding author had full access to all the data in the study and had final responsibility for the decision to submit for publication.

## Results

**Figure 1:**
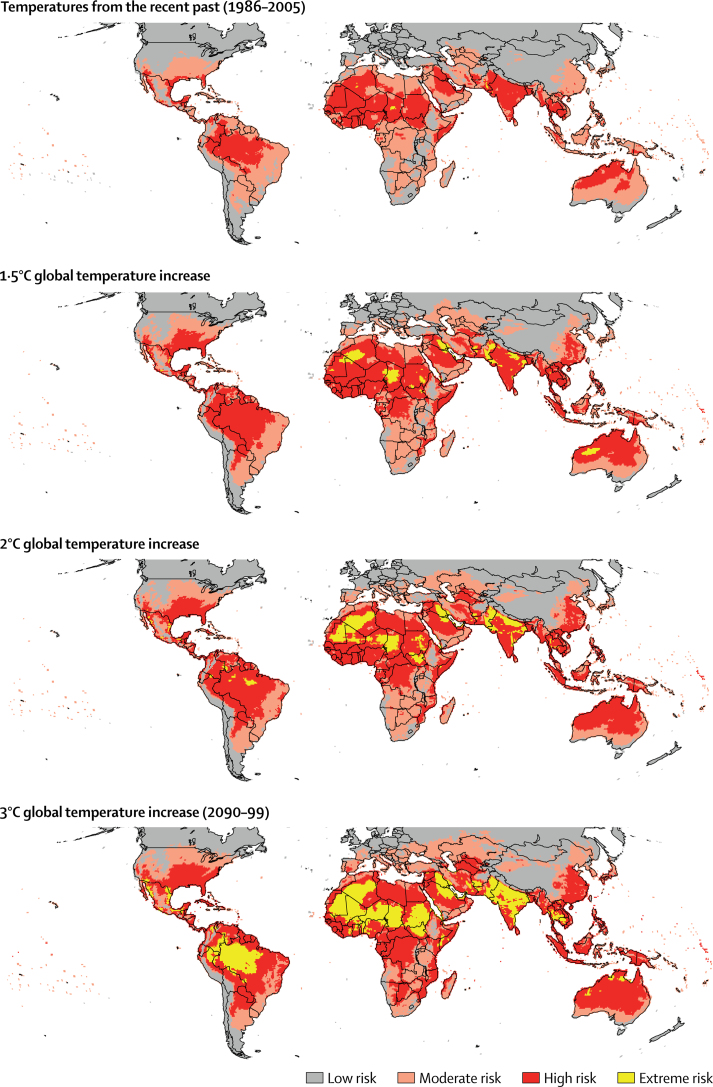
Global risks of occupational heat exposure in the shade during the hottest part of the day, averaged over the hottest month Results are illustrative from an average of four models calibrated to observations. For global temperature increases of 1·5°C and 2°C above pre-industrial levels, the temperature change for individual models is taken from the first decade at which they reach that temperature. For global temperature increases of 3°C, 2090–99 is taken (which is the last decade available). Low risk was defined as a WBGT of 25°C or less; moderate risk was a WBGT of 26–29°C; high risk was a WBGT of 30–33°C; and extreme risk was a WBGT of 34°C or more. Results of individual models, which also show individual climate variability, are shown in the appendix. WBGT=wet-bulb globe temperature.

In the recent past (ie, 1986–2005), the risks of in-shade heat exposure were moderate to high during the warmest part of the day across many tropical and subtropical regions (figure 1). A sensitivity analysis indicated that, in subtropical regions, full-sun conditions could cause a further increase in WBGT of more than 3°C by 2090–99, compared with in-shade exposure (appendix). As such, the following results are likely to be conservative compared with the risks of heat stress in full-sun conditions.

Extreme risk of heat exposure is currently present in southern Pakistan and a few areas of central north Africa (Chad, Algeria). The risk of extreme heat exposure with increasing global temperature in countries with a 2017 population that exceeded 10 million is shown in the panel. If the GTC increases to 1·5°C above pre-industrial levels, the area exposed to extreme heat is projected to expand to include more areas of Africa (eg, Mali and Niger), more areas of south Asia, Iraq and Saudi Arabia in the Middle East, and Australia (although western Australian extreme heat exposure decreases in spatial extent at higher GTCs due to climate variability in models). If the GTC increases to 2°C more than pre-industrial levels, the risk of extreme heat stress extends much further, across many subtropical areas, particularly to regions across much of north and central north Africa, areas of central South America. Increased exposure to extreme heat stress after a 2°C increase in the global temperature is also projected to occur across western and south Asia (including larger areas of Pakistan and India). If the GTC increases to 3°C more than pre-industrial levels, the risk of extreme heat stress would affect a much larger area of South America, particularly Brazil and Peru, much larger areas of south Asia, and tropical and subtropical Africa (including Ghana). The number of countries with more than 10 million people that are classified as at risk of extreme occupational heat exposure more than doubles between an increase in the global temperature of 1·5°C and 3°C.

Panel **Countries with a 2017 population of more than 10 million people that are exposed to an extreme risk of heat stress, in the recent past and after different increases from pre-industrial temperatures**

Data are based on a monthly average of daily maximum shaded wet-bulb globe temperature (in the warmest month) for the recent past (1986–2005) and for increases in global temperatures of 1·5°C, 2°C, and 3°C, and use World Bank data. Duplicate countries with greater increases in global temperature are omitted.

**Currently (by use of data from the recent past)**

• Algeria
• Chad
• India
• Mexico
• Pakistan

**After a 1·5°C increase**

• Afghanistan
• Australia
• Bangladesh
• Cambodia
• Ethiopia
• Iran
• Iraq
• Mali
• Nepal
• Niger
• Saudi Arabia
• South Sudan
• Sudan
• Thailand
• Yemen

**After a 2°C increase**

• Brazil
• Cameroon
• Colombia
• Ecuador
• Guatemala
• Nigeria
• Peru
• Somalia
• Tunisia
• USA
• Venezuela

**After a 3°C increase**

• Burkina Faso
• Benin
• Bolivia
• Ghana
• Côte d'Ivoire
• Kenya
• Myanmar
• Senegal
• Syria
• Vietnam

Comparatively, the areas at risk of moderate and high heat exposure are less sensitive to global temperature increases than the areas at risk of extreme heat exposure; these risks have similar zonal and meridional distributions but show notable expansions in southeast China, the USA, and Australia (figure 1).

Patterns of heat exposure risk (including moderate and high exposure) for individual models are broadly consistent with mean projections across all models (appendix); however, between-model differences highlight the important effects of structural differences and internally generated variability in model projections.

The presence of extreme heat exposure in highly populated regions leads to amplified overall risks to workability and survivability. The number of people who are exposed to WBGTs that are greater than the thresholds for workability and survivability will reflect the interactions between regional exposure and population growth. The use of thresholds means that rapid changes in the exposed population can occur in response to natural climate variability and climate change, as we have modelled and as happens in reality. We have separated urban and rural populations to assess these interactions.

**Figure 2:**
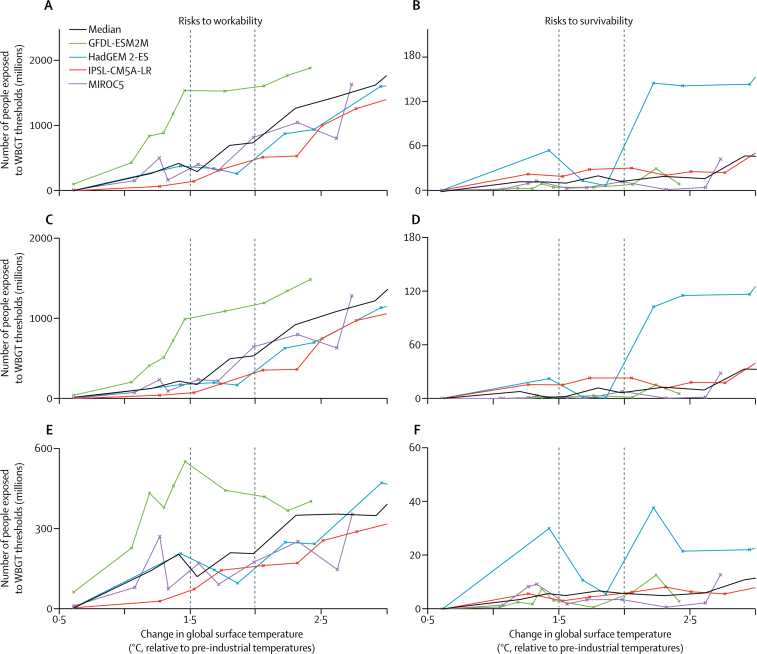
Number of people exposed to heat stress above the risks to workability and survivability thresholds at a given change in global mean surface temperature relative to pre-industrial levels Data are shown for the effects of an increased global surface temperature on risks to workability, overall (A), and in urban (C) and rural (E) areas, and on risks to survivability, overall (B), and in urban (D) and rural (F) areas. Climate and population data are aggregated by decade and exposure reflects the population at the time a given GTC is reached. The workability threshold is crossed when the maximum monthly mean wet-bulb globe temperature exceeds 34°C at the warmest part of the day, and the survivability threshold is crossed when the maximum daily wet-bulb globe temperature exceeds 40 °C for 3 consecutive days. Median number of people exposed across the four models is shown for each decade. The median, rather than the mean, is used because it is more representative of the model ensemble, given the relatively small number of models and the large spread among models. Dotted lines are overlaid at an increase in global temperature of 1·5°C and 2°C.

At a GTC corresponding to a 1·5°C increase, around 350 million people would be exposed to WBGTs exceeding the workability threshold (range 140 million–1·5 billion for individual models). For GTCs of approximately 2·5°C, the number of people exposed to WBGTs in excess of the workability threshold approaches 1 billion people in all models (median of 1·4 billion), consistent with highly-populated tropical and subtropical regions experiencing extreme heat stress (figure 2). Urban populations account for most of the increases in median population exposure to the workability threshold at higher GTCs across several models.

The median population exposure to WBGTs greater than the workability threshold across several models increases as a function of GTC. All models project increases in the number of people exposed to WBGTs greater than the workability threshold of 100 million people or more for some decades as populous low-latitude areas smoothly cross thresholds of persistent extreme heat with increasing GTC (figure 2). However, considerable variability exists across models and over decades. Projections based on one model, the GFDL-ESM2M, indicate an increase of about 1 billion people exposed to extreme heat stress that is greater than the workability threshold for GTCs approaching 1·5°C. Consistent with this result, projections with the GFDL-ESM2M model indicate more geographically widespread risks of extreme heat exposure at increases in global temperature of 1·5°C when compared with other models (appendix). This rapid increase mainly arises when the workability threshold is crossed in urban centres of India and Pakistan. At increases in the global temperature exceeding 1·5°C, projections with the GFDL-ESM2M model indicate a smaller increase in total population exposed and a reduction in rural population exposure due to less geographically widespread extreme heat in rural populations of central north India (appendix). This model behaviour indicates that persistent extreme heat across populous areas of south Asia might be strongly modulated by natural climate variability and how that variability is itself affected by climate change.

Other models also showed considerable variability over several decades in exposure to the workability threshold in south Asia (appendix). For example, the total population who would be exposed to WBGTs greater than the workability threshold increases in one decade by more than 500 million people at GTCs above 2·5°C in the projections based on the MIROC5 model, which is attributable largely to climate shifts on the Indian subcontinent. Therefore, even if the GTC increases relatively smoothly, the presence of a threshold in workability means that the population exposed can change suddenly.

A smaller proportion of the global population is projected to be exposed to the survivability threshold than the workability threshold (figure 2). According to several models, the exposure to the proposed threshold for survivability of heat stress was not reached in the recent past. From the multi-model median projection, the numbers of people exposed to risks to survivability increases with GTC, to reach around 20 million people globally at warming of about 2·5°C. For a mean global temperature increase across models of 3°C, about 50 million people globally would be exposed to WBGT above the survivability threshold, but with a large model uncertainty. Like the findings for risks to workability, populations exposed to a survivability threshold are predominantly located in urban areas. Projections based on the HadGEM2-ES model indicate non-linear increases in population exposure to WBGT greater than the survivability threshold from about 50 million people at increases of 1·5°C in global temperature to about 140 million people at increases of 2·5°C in global temperature. This abrupt increase in one model is associated with expansion of specific at-risk areas in central Africa (including Chad and Niger) and in highly populated areas of northeastern Pakistan and northern India.

Projections with other models indicate smaller increases in population exposure to WBGTs greater than the survivability threshold as a function of the GTC; however, the number of people exposed is largest for GTCs corresponding to increases of more than 2·5°C. With the MIROC5 model, population exposure to heat stress greater than the survivability threshold increases by an order of magnitude to more than 40 million people for increases in global temperature of more than 2·5°C because more populous regions of north Africa are affected. Such associations highlight the sensitivity of threshold behaviours in coupled social-climate systems to projected patterns of population change, particularly in urban areas. All models showed that many millions of people could be exposed to heat stress that exceed thresholds for workability or survivability at increases in global temperature of more than 2·5°C.

## Discussion

By use of physiology-based thresholds for workability and survivability under extreme temperatures, we showed that habitability has the potential to be impeded for some subtropical and tropical areas in response to projected climate change. Extreme heat exposure could affect hundreds of millions of people globally in response to GTCs that would meet international targets. These results agree with the conclusions of a previous study^9^ that used a different approach and did not directly assess thresholds of the survivability of populations. Non-linear interactions between risk of heat exposure and regional climate variability mean that substantial increases in populations exposed to heat stress above workability and survivability thresholds could occur within a decade. For instance, as highlighted by Rohini and colleagues,^29^ future changes in the variability of heat waves, particularly on the Indian subcontinent, might occur in response to projected increases in the sea surface temperature in the tropical Indian Ocean and central Pacific. In other tropical and subtropical regions where heat exposure risk is largest, annual variability in summertime WBGT has been shown to be low.^6^

Risks of heat stress are lowest at lower GTCs; the proportion of the population that is likely to be exposed to WBGTs above workability thresholds is greatly reduced if GTCs remain at less than 1·5°C more than pre-industrial levels, at which point the projected population exposure reaches 350 million people (model range 140 million–1·5 billion). Risks to survivability are also lowest if GTCs remain at less than 1·5°C above pre-industrial levels, compared with a multi-model mean global temperature increase of approximately 3°C in which about 50 million people would be exposed globally; however, the model uncertainty for the number of people exposed to heat stress that would exceed the survivability threshold was large.

Population growth is projected to be highest in the regions at the highest risk of heat exposure (eg, central and west Africa and south and southeast Asia), emphasising the importance of anticipating and preparing for future change with effective adaptation strategies. The largest changes in risks to workability and survivability occur in urban populations, where increases in heat stress could be further amplified by patterns of urbanisation and the associated changes in land use.^30^ Cities tend to be hotter than rural areas through the so-called urban heat island effect, which is not incorporated into these models and which includes heat loading outside buildings and reduced cooling from reduced evaporation. Our estimates of extreme heat exposure are therefore conservative for urban areas in the absence of air conditioning.

Reducing people's capacity to work could increase poverty and inequality in some regions by reducing income or increasing risk of death because of pressure to maintain livelihoods. The ability to work will also be impaired to a considerable extent below the applied workability WBGT threshold and the limitations in work capacity will be particularly pronounced for heavy labour. For poor rural communities, it is also possible that subsistence farmers could be exposed to full-sun outdoor heat stress conditions in the future, which are, on average, 3°C WBGT higher than for the shaded conditions that we have estimated (appendix). In these communities, this full-sun exposure could substantially increase impoverishment.^31^ For affected rural populations, there are few adaptation options because of the need to work outdoors all year and because of the high cost of air conditioning. As such, to combat workplace heat stress in low-income and middle-income countries affected by climate change, new occupational health initiatives and mechanisation will be required.^32^

Several measures could be implemented to reduce the risks of exposure to temperatures greater than workability and survivability thresholds. Such measures include keeping global temperature increases to the minimum possible and recognising that serious risks scale with the level of warming. The options for local adaptation are restricted to active cooling such as air conditioning or changing the work schedule to the coolest parts of the day or to different seasons, provided that these changes are affordable and practical. Millions of people might have no other adaptation option other than migration, either within the country (seasonally or permanently)^22^ or to other countries, particularly when the survivability threshold is exceeded. Anticipating and managing heat stress in hot regions and building on commitments in the Paris Agreement is therefore crucial in responding to climate change.
