# Supplementary material for: Implications for workability and survivability in populations exposed to extreme heat under climate change: a modelling study
Source: Lancet Planet Health. 2018 Dec;2(12):e540–7. doi: 10.1016/S2542-5196(18)30240-7 (PMC6358125; doi:10.1016/S2542-5196(18)30240-7)
Supplement: Supplementary appendix [file mmc1.pdf]

THE LANCET
Planetary Health

Supplementary appendix

This appendix formed part of the original submission and has been peer reviewed. We post it as supplied by the authors.

Supplement to: Andrews O, Le Quéré C, Kjellstrom T, Lemke B, Haines A. Implications for workability and survivability in populations exposed to extreme heat under climate change: a modelling study. *Lancet Planet Health* 2018; **2:** e540–47.

Supplementary Information

Calculation of in shade WBGT

For in shade conditions at constant wind speed (1 m s^{-1}) the standard WBGT formulation is reduced to:

$$WBGT = 0.67T_{pwb} + 0.33T_a \qquad \text{Equation 1}$$

where T_{pwb} is the psychometric web bulb temperature and T_a is air temperature (°C). T_{pwb} is calculated following Jensen [1] (Eq. 2):

$$T_{pwb} = \frac{\gamma T_a + \ \delta T_d}{\gamma + \delta}$$

where,

$$\delta = \ \frac{4098e}{(T_d + 237.3)^2}$$

$$\gamma = 0.00066ps$$

where e is vapor pressure (kPa), ps is barometric surface pressure (kPa) and T_d is dewpoint temperature (°C), calculated following Equation 5:

$$T_d = 243 \ln(\frac{e}{0.6112})/17.67 - \ln(\frac{e}{0.6112})$$

Figure S1. Impact of direct solar radiation on calculated monthly WBGT for the average of end-century (2090 – 2099, HadGEM2-ES). Upper: positive ΔWBGT (° C) values indicate a positive contribution from direct solar radiation. Lower: Zonal mean WBGT (° C), no direct solar radiation (red), direct solar radiation (black). Time varying downwelling shortwave radiation follows Representative Concentration Pathway 6.0 (RCP6.0).

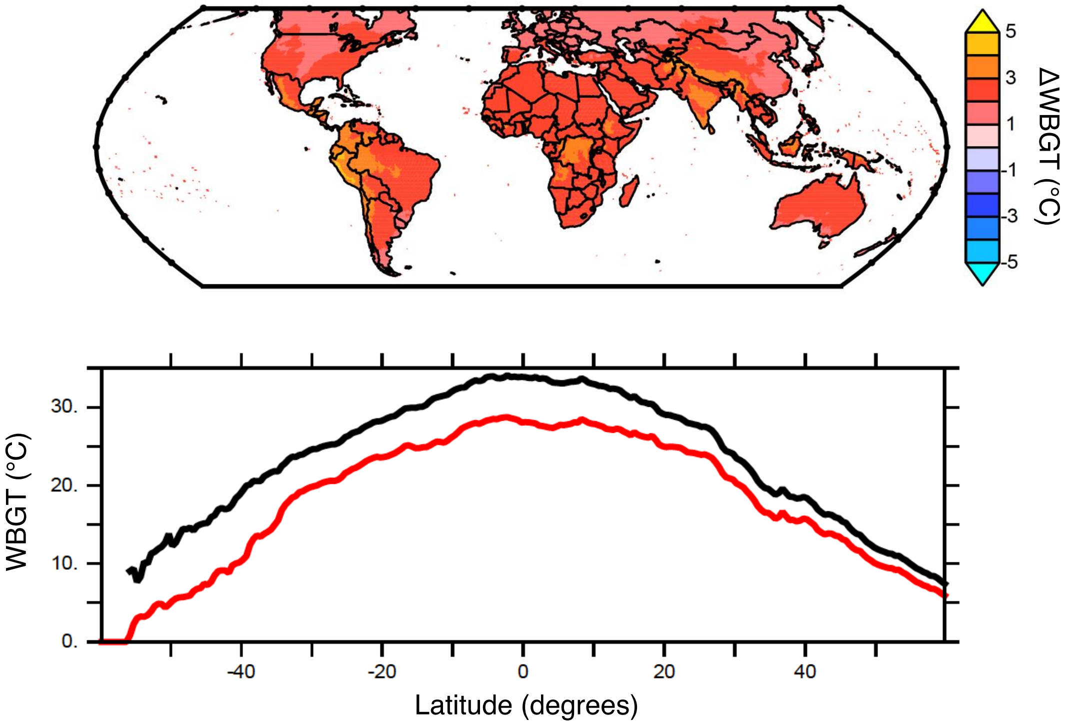

Figure S2. As Figure 1 but for our estimates based on individual ISIMIP2b climate model data. Global Temperature Changes (GTCs) of + 1·5 °C, + 2 °C, 2·5 °C, 3 °C, and + 3·5 °C above pre-industrial as indicated on each panel.

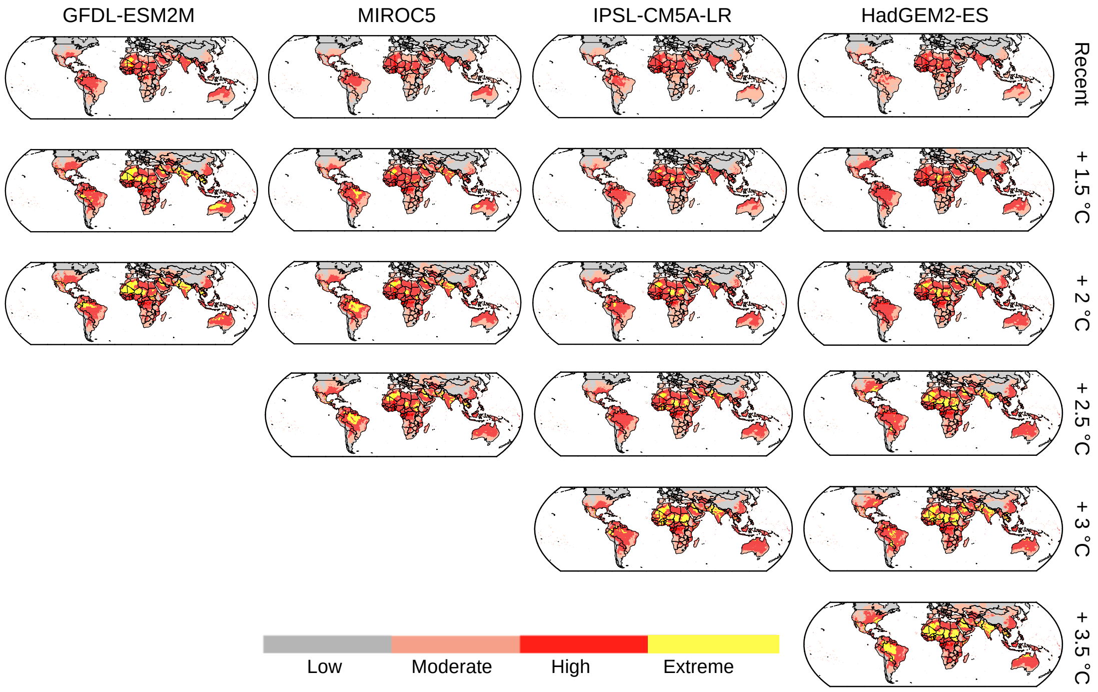

References

1. Jensen, M. E., Burman, R. D., & Allen, R. G. (1990). Evapotranspiration and irrigation water requirements. ASCE.
